# Constitutional Treatment of Diseases of the Female Sexual Organs

**Published:** 1895-01

**Authors:** William Redin Kirk

**Affiliations:** Assistant to the Chair of Gynecology in the Hospital College of Medicine; Visiting Gynecologist to the Louisville City Hospital, etc., Louisville, Ky.


### CONSTITUTIONAL TREATMENT OF DISEASES OF THE FEMALE SEXUAL ORGANS.

By WILLIAM REDIN KIRK, M. D.,
Assistant to the Chair of Gynecology in the Hospital College of Medicine; Visiting Gynecologist to the Louisville City Hospital, etc., Louisville, Ky.

Disorders of the female sexual organs are no exception to the universal rule that all local diseases, whether organic or functional, are largely under the control of general systemic medication. This simple truth in the present operative era of gynecology is often overlooked in the mad desire to establish brilliant surgical statistics, and unwarrantable invasion, for the relief of local conditions, is often practised in the name of science when the cause is far remote, as is generally evidenced by the failure to relieve the symptoms.

It is not my object in this paper to underrate the importance of local medical or surgical interference in those cases which require prompt and effective action, but to call attention to the fact that many of the simple, though none the less aggravating, forms of sexual disorders are greatly benefited and often relieved by judicious constitutional medication.

Bearing in mind the dependence of the sexual organs upon the nutritive and nervous systems for proper nutrition and innervation, it readily becomes apparent the effect that derangement of the general constitution may have upon these special organs, and the relief which can reasonably be expected by directing our agents through these channels, instead of needlessly tinkering with slight local conditions, which have been magnified by the imagination into lesions of serious importance.

The overworked school girl, housed and enervated, complaining of loss of appetite, cold feet, headache, backache and general malaise, will most likely be found suffering from amenorrhea, accompanied by a slight vaginal catarrh and constipation. She needs no local treatment, and it would be outrageous to subject her to the humiliation. The trouble lies beyond, in the central nervous and nutritive systems, which have been sapped of their energy at a time when most needed to support and supply nervous energy to the rapidly developing reproductive organs.

Almost the identical symptoms are found in the over-worked factory girls, who are insufficiently clothed and nourished, and shop girls, who are compelled to stand for hours behind counters, in close, illy-ventilated stores, at times when the sexual system is at its periods of greatest activity.

These symptoms are not confined to the indigent and laboring classes, for nervous, irritable women, who lead sedentary, objectless lives, and that large army of society votaries, who spend their time flitting from one social function to another, wasting their nervous energy on balls, receptions and late suppers at the club, pay the penalty of their folly sooner or later. These form by far the greatest number of cases of dysmenorrhea which fall to the care of the gynecologist.

The loss of sleep and excitement soon fades the beautiful rosy bloom from the debutante's cheek and fills the nights with restlessness and her days with pain and care. She is compelled at each menstrual epoch to seek her couch, for the congested and hypersensitive endometrium cries out with pain. She has headache, backache, pelvic pains, irritable bladder, pain down the thighs and spinal irritation, all due to hyperesthesia of the lining membrane of the uterus, brought about by nervous exhaustion.

What all these cases need is radical change of habits, good wholesome food, plenty of air and sunshine, and general tonic treatment, combined with some agent which has a special predilection for the sexual system.

Such agents are called alterative uterine tonics, and of such agents my choice has long been that very happy combination of uterine alteratives and sedatives called ponca compound; it has a decided effect upon the endometrium. It owes its therapeutical value to the following agents:

Mitchella repens is astringent, diuretic and parturifacient. It also favors the occurrence of menstruation and is an excellent remedy in the treatment of dysmenorrhea and menorrhagia. The characteristic pains of the disorder diminish in intensity and disappear completely under its use. It is also of use in colic and has been found peculiarly efficacious in atony of the female generative organs and in leucorrhea.

Caulophyllin is a general systemic sedative and antispasmodic in its action. It exerts a decidedly alterative influence on the uterus, whence its value in subinvolution and endometritis. It also exerts a similar influence upon the ovaries, whence the benefit resulting from it in amenorrhea and menorrhea. Its antispasmodic action is evident in its influence over hysteria. In threatened abortion and allied conditions it is of value.

Helonias is an excellent general nervine tonic, with a special tendency to the bladder, gastrointestinal tract and pelvic organs. It is of decided value in cystitis, ascites and in amenorrhea, dysmenorrhea and menorrhagia.

Viburnum paralyzes both the centers of voluntary motion and the reflex functions of the spinal cord, without impairing sensation or consciousness, and it is consequently an approved remedy in all diseases characterized by excitability of the motor centers. It has a special relation to the uterine nervous system, has been found especially invaluable in menorralgia, dysmenorrhea, in abortive tendencies, in after-pains, in ovarian irritations and in menorrhagia.

It has long been my habit to prescribe it alone, or in combination with other agents. In the amenorrhea of anemia and nervous exhaustion I find it quite a valuable aid to the regular treatment, and in nervous dysmenorrhea I rely upon it more than the bromides, as it relieves the spasms and has a sedative effect upon the pelvic circulation, without impairing sensibility, or the risk of producing drug habit.

Ponca can be continued with impunity between the menstrual epochs, when bromides, given even to the point of relieving nervousness without producing sleep, can hardly be continued longer than a few days.

---

According to Bruns, six per cent. of patients under ten years of age who have been cured of hip disease, nine per cent. of those between ten and twenty years and seven per cent. of those above twenty years of age, succumb eventually to tuberculosis of other organs.—*Memphis Medical Monthly.*

